# Pathophysiological Aspects of Aging in Venous Thromboembolism: An Update

**DOI:** 10.3390/medicina58081078

**Published:** 2022-08-10

**Authors:** Dimitra Akrivou, Garifallia Perlepe, Paraskevi Kirgou, Konstantinos I. Gourgoulianis, Foteini Malli

**Affiliations:** 1 Respiratory Medicine Department, Faculty of Medicine, University of Thessaly, 41300 Larissa, Greece; 2 Respiratory Disorders Lab, Faculty of Nursing, University of Thessaly, 41300 Larissa, Greece; 3 Respiratory Medicine Department, University Hospital of Larissa, 41223 Larissa, Greece

**Keywords:** aging, elderly, pathophysiological changes, coagulation factors, clinical implications

## Abstract

The aim of this review is to highlight all the factors that associate venous thromboembolism (VTE) with aging. Elderly people are characterized by a higher incidence of thrombosis taking into account the co-existing comorbidities, complications and fatality that arise. Based on the Virchow triad, pathophysiological aspects of venous stasis, endothelium injury and hypercoagulability in elderly people (≥65 years) are described in detail. More precisely, venous wall structure, nitric oxide (NO) and endothelin-1 expression are impaired in this age group. Furthermore, an increase in high-molecular-weight kininogen (HMWK), prekallikrein, factors V, VII, VIII, IX and XI, clot lysis time (CLT) and von Willebrand factor (vWF) is observed. Age-dependent platelet dysfunction and changes in anticoagulant factors are also illustrated. A “low-grade inflammation stage” is delineated as a possible risk factor for thrombosis in the elderly. Consequently, clinical implications for frail elderly people related to diagnosis, treatment, bleeding danger and VTE recurrence emerge. We conclude that aging is an acquired thrombotic factor closely related to pathophysiological changes.

## 1. Introduction

Physiological hemostasis is regulated by multiple factors, which work in harmony, in order to maintain the balance between inhibitors and stimulators of thrombus formation. Factors contributing to venous thrombosis can be categorized in three main clusters as described by Rudolf Virchow in 1856: endothelial injury, hypercoagulability and venous stasis [1]. Aging is associated with comorbidities in the vast majority of the elderly and, thus, advancing age has been causally associated with the three basic pathophysiological mechanisms mentioned above. The scope of this review is to present the impact of physiological aging on the Virchow triad and to decode the mechanisms responsible for the disruption of hemostasis in the elderly (i.e., patients aged ≥ 65 years). The added effect of comorbidities on the pathophysiology of venous thrombosis in the elderly will not be extensively discussed.

## 2. Definition of Aging and Old Age

Venous thromboembolism (VTE) is a major public health issue and has been a subject of research for many years. Pulmonary embolism (PE) and deep-vein thrombosis (DVT) are the two manifestations of VTE. The occurrence and recurrence of VTE are regulated by multiple factors, making prevention and management of patients challenging [2,3,4,5]. VTE affects all ages, with an overall annual incidence in the general population of 117 per 100,000 (95% CI, 112–122) [6]. The risk of a first VTE event varies from 0.91 (95% CI, 0.90–0.91) to 1.43 (95% CI, 1.33–1.54) per 1000 person-years [7,8]. However, these results differ when the study population is divided into age groups, since VTE is uncommon in children and is primarily a disease of older age [6,7,8,9,10,11,12,13,14,15,16]. Studies have reported a steady increase in the annual incidence in the elderly population, in both sexes [6,8,12,13,14,15] as well as in hospitalized patients [14]. In more detail, incidence rates in patients aged ≥65 years are 3-fold higher than in patients aged 45–54 years [13]. Specifically, there is a >7–10-fold increase from ages <55 to >75 years [9]. In addition, the cumulative risk of a first-time VTE event rises from 0.5% at the age of 50 to 10.7% at the age of 80 years [11]. Some variation exists between different ethnic groups and geographical regions [10,17,18,19,20,21] but, interestingly, the age-associated increase in the incidence appears to be universal [19,20].

An increasing body of evidence suggests that VTE fatality is elevated in the elderly [7,8,15,22]. Thirty-day case-fatality rates after first VTE event vary from 6.4% (95% CI, 4.6–8.1) to 14.10% (95% CI, 13.84–14.36) [7,8,13] and, specifically, there is a 3-fold increase from ages 40–49 to ≥80 years [8]. In the same context, the 1 year mortality rate varies from 21.6% (95% CI, 18.7–24.8) to 29.21% (95% CI, 28.86–29.55) [7,8] and there is a 2.5-fold increase from ages 40–49 to ≥80 years [8]. Results from studies in the elderly population have shown a mortality rate of 9.9%, 28.4%, and 37.0% at 30-day, 1-year, and 3-year follow-up, respectively [23]. There are similar results among hospitalized elderly populations [14,24]. Differences between sexes were noted in some studies [7,8,22], with men presenting a higher mortality rate than women. Regarding the factors that may contribute to the age-dependent increase, one has to take into account malignancy, cardiovascular diseases, neurological impairment (with paresis or paralysis) and major abdominal surgery [7,8,13,15,23,24,25].

As far as recurrence of VTE is concerned, results vary depending on the underlying cause of the event and management of the patient [26]. Recurrence seems to have a time-dependent increase [23,27], which can reach 30% at 10 years following the first event [28]. Men have a significantly higher risk of recurrence, with a relative risk (RR) of 1.6 (95% CI, 1.2–2.0) compared to women [29]. Cancer has also been associated with an elevated risk [13,25,28]. Older cancer patients experience a higher recurrence risk [28]. However, the effect of increasing age as a predictor of recurrence is still debated with studies providing conflicting results [13,23,27,28,30,31]. In prospective studies, older age is associated with increased recurrences following a first event of VTE [13,28], while others have challenged this finding [23].

Increasing age, current or recent hospital or nursing home confinement, malignancy with or without chemotherapy, congestive heart disease, trauma, surgery and chronic renal disease are the major acquired risk factors of VTE [2,3,4,7,8,10,13]. VTE events can be classified by their underlying cause in two major categories; provoked/secondary and unprovoked/idiopathic [2]. In some studies, secondary cases are further distinguished in cancer and non-cancer related [7,8,13]. VTE in the elderly is mostly provoked (45%) [23], with malignancy-related events accounting for 26% of overall events [23]. In comparison to younger age groups (≤75 years), VTE events are more likely to be associated with immobilization (OR: 2.46, 95% CI, 1.85–3.27) and with severe medical disorders (OR: 1.99, 95% CI, 1.41–2.80) [32].

## 3. Pathophysiological Impact of Aging on VTE

**Figure 1:**
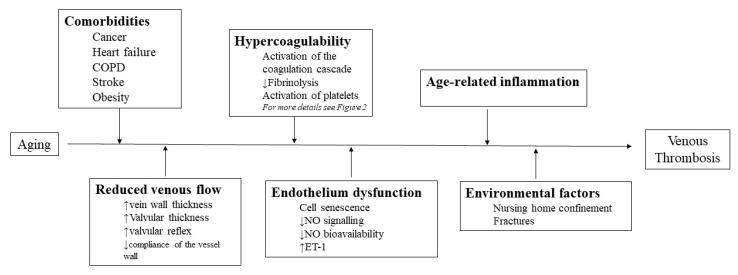
The main mechanisms underlying the impact of aging in VTE. Some of the factors are interrelated (see text for details). ↑: up-regulation, ↓: down-regulation.

Figure 1 summarizes the main mechanisms underlying the pathophysiological impact of aging in VTE.

### 3.1. Venous Stasis

The term venous stasis includes all changes occurring in the blood flow from the peripheral veins to the heart, varying from decrease to stasis [33]. Hemodynamic alterations in blood flow can result from venous wall and/or valve incompetence or immobility-related factors [34,35,36,37].

Chronic venous insufficiency is a common condition in older patients [37,38] and has been associated with up to a 3-fold elevated risk of venous thrombosis (DVT and PE) [36]. The pathogenetic mechanism underlying this appears to be complex; changes occur both in the venous valvular properties and the vessel walls [37]. Specifically, venous valves and walls undergo structural changes with increasing age [39]. This has been discussed in the literature since the 1950s, when Saphir and Lev described histological changes in the iliac, popliteal and femoral veins that occurred after the third decade of life [39,40]. These findings seem to correspond with the hemodynamic disturbance that takes place in the elderly [39,40,41]. Interestingly, in the 1960s, a study presented a prolonged clearance time of contrast during venography in older patients, which was associated with a clear gradient decrease in venous blood flow [42]. Additionally, one has to take into account that in recent years, research has focused on hemodynamics of the venous system; a significant decline in the diameter, flow velocity and flow of the common femoral vein exists in subjects over 70 years, compared to younger age groups [41].

Valvular thickness plays a major role in VTE, with measures above the than 90th percentile accounting for a 3-fold increase in the risk [43]. Accumulated data suggest that aging is associated with fibrosis and thickening of the vein wall and the valve cusps and reductions in the compliance of the vessel wall [33,39,44,45]. Mean valve thickness ranges from 0.35 mm in 20–30 years to 0.59 mm in subjects 71 to 80 years that accounts for 0.004 mm increase per year [46]. Valvular function appears to be inversely related to valve thickness. Furthermore, thickness is related to the closing time of the valve. Delayed closing of the valve results in valvular reflux, which is associated with a 2.8-fold increase in the risk of VTE in the elderly [43]. Furthermore, is frequently a significant problem in the older subjects. Immobility-related risk factors of VTE include hospitalization, surgery, fractures, plaster cast, minor leg injury and transient immobility at home [4]. These factors are strongly associated with the occurrence of VTE in the elderly, with a 1.9–14.8-fold increase in the risk [4].

All changes mentioned above result in blood flow disturbance and, specifically, in vortical flow in the valve sinus. Thus, the microenvironment of the sinus is further disturbed, creating hypoxia and, subsequently, activation of the endothelium and the coagulation cascade [35,47,48].

### 3.2. Endothelium Dysfunction

Endothelial dysfunction (ED) plays a key role to venous thrombosis and aging is a contributing factor to its occurrence [49]. The pathophysiology of aging’s effect on the ED includes endothelium cell senescence [50]. Cell senescence describes the cessation of cell proliferation accompanied by phenotypical changes; more specifically, endothelial cell senescence has been associated with increased risk of cardiovascular diseases [50,51]. The pathophysiological pathway leading from cell senescence to thrombosis is multifactorial [51,52,53,54,55,56].

Nitric Oxide (NO) plays a crucial part in endothelial function, as well as in the regulation of hemostasis [57]. As a result, it is a major contributor to ED [49]. Although NO synthase expression is stable irrespective of age, NO vasodilation signaling is impaired in aging [57], resulting in disturbance of the vascular tone [52]. The underlying cause of NO signaling impairment appears to be the chronic oxidative stress that accompanies older age [53]. Furthermore, mitochondrial biogenesis of endothelial cells decreases with age, causing an elevation in reactive oxygen species and a reduction in NO bioavailability [52]. Thrombospondin-1, Thioredoxin-Interacting Protein, Symmetric and Asymmetric Dimethylarginine and Myeloperoxidase have also been described as factors contributing to impaired NO signaling [57]. As far as hemostasis is concerned, NO acts a platelet antiaggregating factor. However, the platelets’ response to NO signaling also declines with age [57]. This results in an imbalance of the proaggregants and antiaggregants and, eventually, enhances platelet aggregation [57].

Endothelin-1 (ET-1) has a similar role, acting upon both vascular tone and platelet function. On the contrary to NO, ET-1 has vasoconstrictor properties; as their levels increase with age, the tone of the blood vessels increases too. ET-1 has a double effect on platelet function depending on the receptor it binds with; endothelin receptor A contributes to activation of platelets, whereas endothelin receptor B has an inhibitory role. With advancing age, receptor A activity increases and receptor B activity diminishes, thus reinforcing platelet activation [52]. Studies suggest that post-PE ET-1 levels are elevated, in comparison to controls [58].

Platelet activation is a fundamental part of the formation of venous thrombi. Changes in the vascular tone, resulting from NO and ET-1 changes, as discussed earlier, further intensify the procoagulant effects in the veins [52,57,59,60].

As mentioned in the Section 3.1, hemodynamic changes in the venous valve sinus create hypoxia, which results in activation of the coagulation cascade and endothelial damage [33]. Furthermore, endothelial dysfunction can potentially amplify the effects of hypercoagulability which results from aging [52].

### 3.3. Hypercoagulability

**Figure 2:**
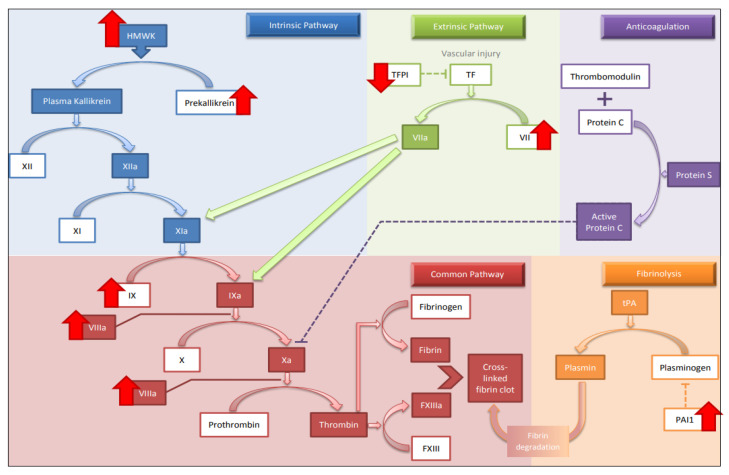
Main changes in the coagulation cascade associated with aging. Abbreviations: HMWK, high-molecular-weight kininogen; TFPI, tissue factor pathway inhibitor; TF, tissue factor; tPA, tissue plasminogen activator. ↑: up-regulation, ↓: down-regulation.

Hypercoagulability was initially introduced in 1998 and is a term used to describe changes in the hemostatic system, including procoagulants, anticoagulants, fibrolytic factors and platelet function. hypercoagulability results in disturbed balance between prothrombotic and antithrombotic factors [61,62,63,64]. All changes mentioned in detail below are displayed in Figure 2.

#### 3.3.1. Extrinsic, Intrinsic and Common Pathway

Altered levels of procoagulants in aging have been a topic in the literature since 1977, when Maede et al. described such changes in fibrinogen [62]. Later on, more studies supported these findings [65,66,67,68,69,70] and some of them shed light to the topic with findings on an age-dependent increase in high-molecular-weight kininogen (HMWK), prekallikrein, factors V, VII, VIII, IX and XI [65,66,67,68,69,70,71].

In the Third Glasgow MONICA survey, in which the coagulation activation markers were studied, the increase measured in factors VII, VIII and IX was associated with elevated activation markers, thus increased activation of the coagulation cascade [67]. Elevated levels of factor VII, VIII and fibrinogen have been previously described as a risk factor for the occurrence of venous thrombosis [72,73,74]. Interestingly, Klovaite et al. observed that elevated fibrinogen levels are associated with a higher risk of PE with DVT [multivariable-adjusted OR: 1.9 (1.0–3.6)] while not associated with isolated DVT [75]. Apart from changes in quantity, aging exerts qualitative changes in fibrinogen; increasing age is associated with structural changes that may contribute to thrombosis [76]. Factor XI deficiency seems to have a protective role against the occurrence of VTE and high levels of factor XI have been established to be a risk factor for VTE [77]. Furthermore, Gallimore et al., found a significant increase in HMWK in patients with VTE compared to healthy donors [78].

Tissue factor (TF) regulation is influenced by increasing age. Specifically, tissue factor pathway inhibitor (TFPI) levels decrease, resulting in reduced inhibition of TF [79]. Deficiency of TFPI has been associated with thrombosis [80,81]. Interestingly, TFPI-1 levels have been found to be a significant marker for the occurrence risk of DVT in patients with non-small-cell lung cancer [82].

#### 3.3.2. Fibrolytic Mechanism

A decrease in the fibrolytic capacity has been established as a risk factor for venous thrombosis [83] and has been proven to be depicted by elevated clot lysis time (CLT) [84]. CLT is determined by plasminogen activator inhibitor-1 (PAI-1), thrombin activatable fibrinolysis inhibitor (TAFI), plasminogen, thrombin and α2-antiplasmin [84].

PAI-1 has a significant impact in fibrinolysis regulation [85,86]. Previous studies have described that PAI-1 levels undergo changes with cell senescence and the physiological aging process [62,63,64,87,88,89]. These changes correspond to the decreased sufficiency of the fibrinolytic mechanism that comes with aging [84,86,90]. The role of increased PAI-1 levels in the formation of thrombi has been a topic in literature since 1961, with Nilsson et al. further supporting their theory with a case–control study in 1985 [91,92]. Since then, many more related studies have been published posing PAI-1 as a potential risk factor for vasculature diseases [83,84].

TAFI is a protein that reflects fibrinolytic insufficiency [84]. An age-related elevation of TAFI levels has been previously described [63] and increased TAFI levels (above the 90th percentile) are associated with a 2–4-fold risk of VTE [84]. Furthermore, a 2-fold risk of VTE recurrence was found in patients with high levels of TAFI in comparison to those with lower levels [93].

Aging’s effect on plasminogen levels is less well described. In more detail, Hamilton et al. found no significant difference in plasminogen among different age groups [94]. However, there was a decrease in plasminogen levels in ages over 85 years [94]. Reduced levels of plasminogen have been associated with an OR of 1.6 (95% CI, 1.2–2.2) for VTE [84].

#### 3.3.3. vWF

Von Willebrand factor (vWF) has a double role in coagulation; it binds platelets with endothelial and subendothelial cells and carries and protects factor VIII from proteolysis [95]. As far as aging is concerned, vWF levels have been found to increase with age in both sexes [96,97]. In recent years, vWF has been under investigation as a risk factor for VTE with contradicting results due to vWF correlation with FVIII [74,98,99]. Despite the results of the early study of Koster et al. [72], there are more recent data that support the notion that increased levels of vWF are independently associated with the occurrence of VTE [98,99]. Interestingly, there is a dose-dependent relationship with HR reaching 7.6 (95% CI, 3.1 to 18) in individuals with vWF levels in the highest fifth percentile [98]. Furthermore, when combined with a major illness and immobility, high vWF levels are associated with an OR of 88.0 (95% CI, 33.9–228.3) [99].

The age-dependent change in the vWF levels has been attributed to the fact that vWF is an acute-phase protein released by activated endothelial cells [100]. Thus, cellular senescence mentioned in the Section 3.2 and endothelial activation due to hypoxia mentioned in the Section 3.1 of the present article could explain the mechanism of elevation of vWF.

#### 3.3.4. Platelet Function

Platelets are a structural part of the thrombus and thus platelet aggregation poses a significant role in VTE [101]. There are several factors contributing to the activation of platelets, some of which undergo changes with increasing age.

First of all, there appears to be a lower threshold for the aggregation of platelets with increasing age [102,103,104]. In addition, various changes in the platelet’s receptors occur with aging. Prostacyclin (PGI_2_) receptors are decreased and, thus, there is a resistance created to PGI_2_-mediated inhibition of platelets [105,106]. Similarly, the affinity of α2-adrenergic platelet membrane receptors, which mediates the responsiveness of the platelets to epinephrine inhibition, is reduced in elderly subjects [107,108]. As mentioned in Section 3.2, changes occur in ET-1 receptors as well [52]. Specifically, ET-A activity, which contributes to activation of platelets, increases with age, whereas ET-B, which has an inhibitory role, diminishes. An age-dependent decrease in NO signaling amplifies the already-existing platelet aggregation [59,60]. All these changes could possibly be depicted, in everyday clinical practice, by changes in bleeding time, which has previously been found to decrease with age [109,110]. Platelet aggregation and reactivity has been found to be enhances by high levels of fibrinogen [111,112], which have been previously mentioned to be affected by age.

#### 3.3.5. Coagulation Factors with Anticoagulant Properties

Changes in the anticoagulant proteins occurring in the elderly has been a topic in the literature since 1977 [113]. Both levels and activity of antithrombin III (ATIII) and protein C (PC) have been under investigation over the years with varying results [67,70,71,96,114,115,116,117,118,119], which are summarized in Table 1 and Table 2, respectively. In more detail, Hager et al. noted a decrease in ATIII levels in the elderly [71]. In the same context, Meade et al. observed that ATIII levels decrease with age in males, but show no change in females [113]. However, Dolan et al. demonstrated that there is a significant age-dependent increase in ATIII only in females [114].

Activity of ATIII appears to decrease with age, which can be depicted by the age-dependent increase in fibrinopeptide A (FPA), a peptide formed by cleavage of fibrinogen by thrombin [71,115]. Nevertheless, Bauer et al. demonstrated that the levels of ATIII are not influenced by age but aging is associated with reduced ATIII activity [115]. Several studies do not replicate the aforementioned results when adjusted for sex (Table 1). Finally, protein S (PS) is not as widely studied as ATIII and PC. Lowe et al. found that PS levels increase with age only in females [67]. However, it has been speculated that PS levels are not associated with the occurrence of VTE, thus PS levels may not fall into our field of interest in the present review [120].

### 3.4. Impact of Age-Related Inflammation

The association of aging and inflammation has been extensively investigated. Aging is accompanied by the development of low-grade systemic inflammation, termed ‘inflammaging’, characterized by raised serum C-reactive protein (CRP) and pro-inflammatory cytokines. Importantly, inflammaging is implicated in the pathogenesis of several of the major age-related diseases including cardiovascular disease, type 2 diabetes, and dementia and is associated with increased mortality.

Aging is considered a low-grade inflammatory state that is termed “inflammaging” [121,122]. Chronic inflammation associated with increased age is characterized by increased pro-inflammatory cytokines and is attributed to comorbidities, adiposity and decreased physical exercise, among other factors [123]. A working hypothesis proposes that low-grade inflammation may be the result of the release of damage-associated molecular patterns (DAMPs), and high-mobility group box 1 (HMGB1) [124]. Accumulated data suggest that age-related inflammation contributes to the development of diseases that occur commonly in the elderly, such as type 2 diabetes and atherosclerosis [125]. Evidence in the literature support that inflammation, innate immunity and hemostasis are interrelated [126]. Studies have shown that age-related inflammation enhances thrombosis by increased levels of tissue factor and platelet reactivity and decreases in thrombomodulin as well as inhibition of fibrinolysis through the increase in PAI-1 [127].

## 4. Clinical Considerations in Elderly Patients Suffering from VTE

Differential diagnosis of pulmonary embolism in the elderly is challenging as associated symptoms (dyspnea, respiratory failure and thoracic pain) are signaling multiple underlying causes that arise from common comorbidities such as heart failure, valvular disease, cancer, neuropsychiatric disorders [128,129]. Furthermore, chronic renal failure, which is typically widespread among elderly [130], is limiting diagnostic accuracy because patients are not able to undergo CT pulmonary angiogram (CTPA) that is the gold standard for PE diagnosis [131]. Thus, numerous questions regarding alternative diagnostic tools and treatment are emerging. Finally, pre-test probability for VTE is also higher in elderly people, as age ≥65 years are scored in the revised Geneva clinical probability test [132].

The treatment of VTE in older adults is complex since they are at increased risk of both thrombosis and bleeding. VTE, and especially PE, is a potentially lethal disease and once it is diagnosed, anticoagulant therapy is promptly initiated if no contraindications exist [133]. No different guidelines exist concerning VTE diagnosis or therapy in elderly subjects. However, in the elderly, VTE diagnosis may pose particular problems since PE may mimic several conditions that are frequent in the geriatric population (e.g., pneumonia). Additionally, d-dimers sensitivity is decreased in older subjects (by approximately 10% in patients aged > 80 years) [134]. To increase sensitivity, studies have suggested the use of an age-adjusted d-dimer cut-off (age × 10 g/L for patients >50 years) [135].

Regarding VTE therapy, researchers have demonstrated that although guidelines do not recommend discrimination of therapy according to age, in real-world data, thrombolysis is less often applied in subjects >60 years [136] suffering from unstable PE, probably due to the increased risk of bleeding in this population [137]. In stable patients, suffering from a PE, anticoagulation is indicated and should promptly be initiated once the diagnosis has been established. Nevertheless, physicians are reluctant to offer anticoagulant therapy in elderly patients [138]. A meta-analysis of observational studies of subjects >75 years suggests that direct oral anticoagulants versus vitamin K antagonists have similar effectiveness concerning VTE recurrence, lower intracranial hemorrhages but more frequent gastrointestinal hemorrhage in the ages group > 75 years [139]. Dose adjustment according to renal function that is frequently impaired in geriatric patients is recommended [140]. The increase in hemorrhagic complications on the elderly may be underlined by the comorbidities (i.e., liver or gastrointestinal disease, renal failure, and or concomitant drug use) [141].

## 5. Conclusions

Increased age is considered a risk factor for thrombosis and aging can be presumed as an acquired thrombophilic state. The mechanism underlying this association includes hypercoagulability, endothelial senescence and venous stasis along with increased chronic inflammation. Additional factors including comorbidities that are commonly present in the elderly (i.e., cancer, chronic heart failure, and stroke) may significantly affect the prothrombotic tendency of older individuals. All of the aforementioned factors are possibly interrelated and result in the increased risk of VTE associated with older age.

## Figures and Tables

**Table 1:** Age-related changes in antithrombin III.

Study (Year)	Antithrombin III Levels	Antithrombin III Activity
**Maede (1977)** [62]	Decrease with age—♂ No change with age—♀	-
**Bauer (1987)** [115]	No change with age *(male subjects only)*	Increase in FPA levels with age, thus decrease in ATIII activity
**Hager (1989)** [71]	Decrease with age	Significant increase in FPA levels with age
**Tait (1993)** [117]	-	Significant decrease with age—♂ Increase with age—♀
**Dolan (1994)** [114]	♂ > ♀ Increase with age—♀	
**Conlan (1994)** [116]	Significant difference ♀ > ♂	Decrease with age—♂ Increase with age—♀
**Amin (2012)** [70]	-	Decrease with age

Abbreviations: FPA: fibrinopeptides A, ♀: female, ♂: female.

**Table 2:** Age-related changes in protein C.

Study (Year)	Protein C Levels	Protein C Activity
**Bauer (1987)** [115]	No change with age *(male subjects only)*	Significant increase in PCP levels with age, thus increase in PC activity
**Hager (1989)** [71]	No change with age	-
**Tahara (1991)** [118]	No change with age—♂ Increase with age—♀	No significant difference—♀ ♂
**Conlan (1993)** [121]	♀ > ♂ No significant change with age	-
**Tait (1993)** [117]	-	Significant increase
**Dolan (1994)** [114]	♂ > ♀ Significant increase with age	-
**Lowe (1997)** [67]	Increase with age—♀	**-**

Abbreviations: PC: protein C, PCP: protein C peptides, ♀: female, ♂: female.

## Data Availability

Not applicable here.
